# Extended-spectrum beta-lactamase (ESBL) producing Enterobacterales in stool surveillance cultures of preterm infants are no risk factor for necrotizing enterocolitis: a retrospective case–control study over 12 years

**DOI:** 10.1007/s15010-020-01453-0

**Published:** 2020-05-27

**Authors:** Martin Eberhart, Andrea Grisold, Michela Lavorato, Elisabeth Resch, Andreas Trobisch, Bernhard Resch

**Affiliations:** 1 Research Unit for Neonatal Infectious Diseases and Epidemiology, Medical University of Graz, Graz, Austria; 2 D&R Institute of Hygiene Microbiology and Environmental Medicine, Medical University of Graz, Graz, Austria; 3 Policlinico Umberto I Hospital, Sapienza University of Rome, Rome, Italy; 4 Division of Neonatology, Department of Paediatrics and Adolescent Medicine, Medical University of Graz, Auenbruggerplatz 34/2, 8036 Graz, Austria

**Keywords:** ESBL, Enterobacterales, Epidemiology, Necrotizing enterocolitis, Preterm infant, Risk

## Abstract

**Purpose:**

Microbial dysbiosis has been found preceding necrotizing enterocolitis (NEC) in preterm infants; thus, we aimed to investigate whether there is evidence that neonates with extended-spectrum beta-lactamase-producing Enterobacterales (ESBL-E) positive stool cultures are at higher risk for NEC at the NICU.

**Methods:**

We included very preterm inborn infants of ≤ 32 weeks of gestational age being fecal carriers of ESBL-E and compared them with 1:1 matched (gestational age, birth weight, gender and year) controls tested negative for ESBL-E in the stool between 2005 and 2016. An association with NEC was defined as the first detection of ESBL-E before or at the time of definite diagnosis of NEC.

**Results:**

During the study period, we diagnosed 217 infants with a total of 270 ESBL-E. We identified ten different species with ESBL-producing *Klebsiella oxytoca* being the most common one (46%) followed by Klebsiella pneumoniae (19%), and *Citrobacter freundii* (17%). Ten out of 217 infants had any kind of NEC in the case group compared to two of the controls (*p* < 0.01), but only four cases with predefined criteria were associated with NEC ≥ stage IIa (1.8 vs. 0.5%, *p* = 0.089, OR 4.1, CI95% 0.45–36.6). NEC mortality rate was 2/8 (25%).

**Conclusions:**

We observed a threefold increase of ESBL-E in stool surveillance cultures during study time and germs were dominated by ESBL-producing Klebsiella spp. There was no evidence that preterm infants colonized with ESBL-E in the stool were at higher risk for definite NEC.

## Background

Extended-spectrum beta-lactamase (ESBL) producing Enterobacterales (ESBL-E) are a growing issue worldwide in health care systems and especially in intensive care units [1]. Intestinal colonization with ESBL-E is regarded as a kind of dysbiosis of the neonatal microbiome and might be associated with the development of necrotizing enterocolitis (NEC) [2, 3]. In particular, invasive infections with ESBL producing *Klebsiella pneumoniae* resulted in high mortality rates [4]. Therefore, surveillance of nosocomial infections at the Neonatal Intensive Care Unit (NICU) is mandatory and hygiene measures like cohort isolation on the NICU and use of gloves, face masks, and coats are necessary as is the implementation of surveillance systems [5].

Necrotizing enterocolitis (NEC) is—despite significant advances in neonatal intensive care of preterm infants—a complex and potentially devastating disease with high morbidity and mortality. Its etiology is by far not completely understood and specific treatment strategies are lacking; surgery is often warranted and short bowel syndrome remains a frightful long-term consequence. The intestinal mucosa of the premature infant presents a border zone that remains in a persistent equilibrium state of either injury or repair [6–10]. It has been recently suggested that NEC is associated with a marked inhibition in both enterocyte migration and proliferation [6]. This leads especially in preterm infants to further injury and finally to bacterial translocation. During these processes, bacteria are able to attack the innate immune defensive system and invade the intestinal epithelial layer with subsequent inflammation and tissue necrosis. During pregnancy so-called Paneth cells appear in the intestinal crypts in the first trimester [10]. Paneth cells represent an important defensive mechanism of the innate immune system since they produce multiple antimicrobial peptides and proinflammatory mediators. By exposure to *K. pneumoniae* in a Paneth cells depleted rat model intestinal injury was initiated and subsequent inflammation was induced that resembled the histopathologic picture of NEC [10]. Infections with ESBL-E in patients are associated with increased morbidity and mortality [11–13]. Our hypothesis was that an altered gut microbiome due to ESBL-E dominated by Klebsiella spp. might increase the risk of developing NEC by the above describes pathomechanisms.

By means of a retrospective single-center observational study (STROBE—Strengthening the Reporting of Observational Studies in Epidemiology statement; https://www.strobestatement.org compliant) [14] and a matched case–control study we aimed to determine the epidemiology of ESBL-E and investigate a possible association with NEC in very preterm infants.

## Methods

Inclusion criteria comprised very preterm inborn infants of ≤ 32 weeks of gestational age being fecal carriers of ESBL-E during the period of 2005 to 2016. These infants were matched 1:1 with very preterm inborn infants of ≤ 32 weeks of gestational age tested negative for ESBL-E in the stool for analysis. Stool collection for these surveillance cultures was done twice a week (Monday and Thursday) throughout the study period in all NICU patients.

Exclusion criteria were severe congenital malformations, genetic disorders and dysmorphic syndromes, and death within the first week of life. Matching criteria included birth year, birth weight (± 100 g), gestational age (± 1 week), and gender (the matching person was blinded for all parameters despite matching criteria; therefore a bias seems very unlikely). Approval of the study was provided by the local ethics committee (EK 29-527 ex 16/17).

ESBL-E testing was carried out by using the chromeID™ ESBL agar plate (bioMérieux, Marcy-l’Etoile, France). Bacterial isolates from the NICU, in general, had been tested for antibiotic susceptibilities in the routine microbiology laboratory using the disk diffusion method or the VITEK 2 system (bioMérieux, Marcy l’Etoile France); ESBL confirmation and interpretation of results were performed as described previously [15].

Data of patients were evaluated from the electronic data management system of the hospital called openMedocs^©^ and the patient data monitoring system called PDMS (Sanofi^©^) of the NICU. Data were extracted and collected using Microsoft Excel^©^. Perinatal data included date of birth, gestational age in weeks, gender, birth weight in grams, Small for Gestational Age (SGA, birth weight < 10th percentile), cesarean section, multiple pregnancies, maternal age, Apgar scores at 1, 5 and 10 min and umbilical artery pH. Neonatal diagnoses included NEC, early-onset sepsis (EOS), late-onset sepsis (LOS), respiratory distress syndrome (RDS), intraventricular hemorrhage (IVH), periventricular leukomalacia (PVL), bronchopulmonary dysplasia (BPD), retinopathy of prematurity (ROP), spontaneous intestinal perforation and ileus.

The diagnosis of NEC was done according to the modified Bell′ criteria and was definite at stage ≥ IIa [16]. In detail the infant had to have abdominal distension, residual gastric volumes, bloody stools, eventually bilious vomiting, and radiologic signs of pneumatosis intestinalis. An association with NEC was defined as the first detection of ESBL-E before or at the time of definite diagnosis of NEC in association with surveillance cultures done every 4 days (see above). The study was done according to the STROBE protocol (supplemental file). During the study period all preterm infants received enteral probiotics (*Lactobacillus casei rhamnosus*), enteral gentamicin, and enteral antimycotic agent (nystatin) as triple anti-infective prophylaxis against NEC [17]. ESBL-E positive infants were separated from ESBL-E negative neonates or isolated as far as possible and cared for using gloves, coats, and face masks.

Blood culture positive EOS and LOS were defined as the presence of three or more out of five clinical signs of sepsis (1) respiratory symptoms (apnea, tachypnea, retractions, cyanosis, respiratory distress); (2) cardiocirculatory symptoms (tachy- or bradycardia, arterial hypotonia); (3) neurological symptoms (lethargy, irritability, seizures); (4) hypo- or hyperthermia (core temperature > 38.5 °C or < 36.0 °C); (5) poor skin color or prolonged capillary refilling time > 2 s) with a pathogen most likely to be causative for sepsis with either maternal risk factors (preterm premature rupture of membranes), chorioamnionitis, and fever during labor) or a positive laboratory sepsis screen with two or more out of four laboratory parameters positive (white blood cell count and absolute neutrophil count with age-adapted cut-off values [18], C-reactive protein > 10 mg/L, immature to total neutrophil ratio > 0.2) and treatment with antibiotics for at least 7 days. EOS was treated with ampicillin combined with cefuroxime during the study period. LOS was treated with teicoplanin and/or imipenem. The average duration of antibiotic treatment was 8.8 days per episode of infection. Rates of EOS and LOS did not change over time.

Statistical analyses were done using Excel^©^ (Microsoft Office, Excel 2013) and SPSS^©^ (IBM SPSS Statistics 22). For categorical data the chi-square test and for numerical data the *t* test was used. The normality assumption was checked using the Shapiro–Wilk test. Odds ratio and 95% confidence intervals were calculated using the software package CIA (Confidence Interval Analysis; version 2.0.0: Statistics with confidence; London: BMJ Publishing Group, 2000). Statistical significance was set at *p* < 0.05.

## Results

**Fig. 1:**
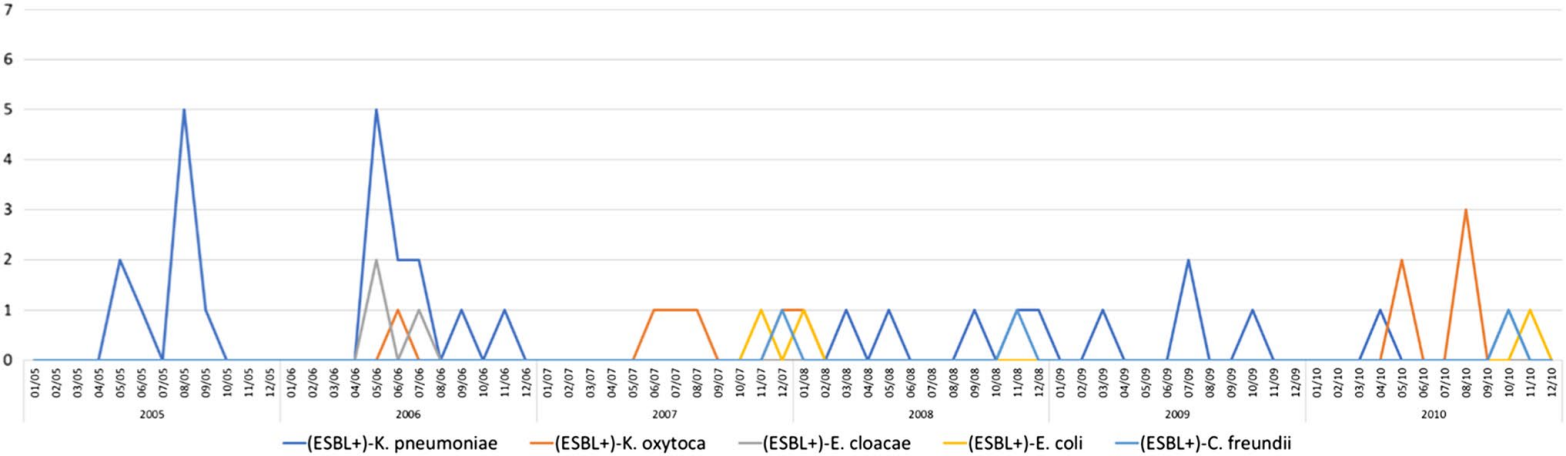
ESBL producing Enterobacterales in surveillance stool cultures of preterm infants ≤ 32 weeks of gestational age detected between 2005 and 2010

**Fig. 2:**
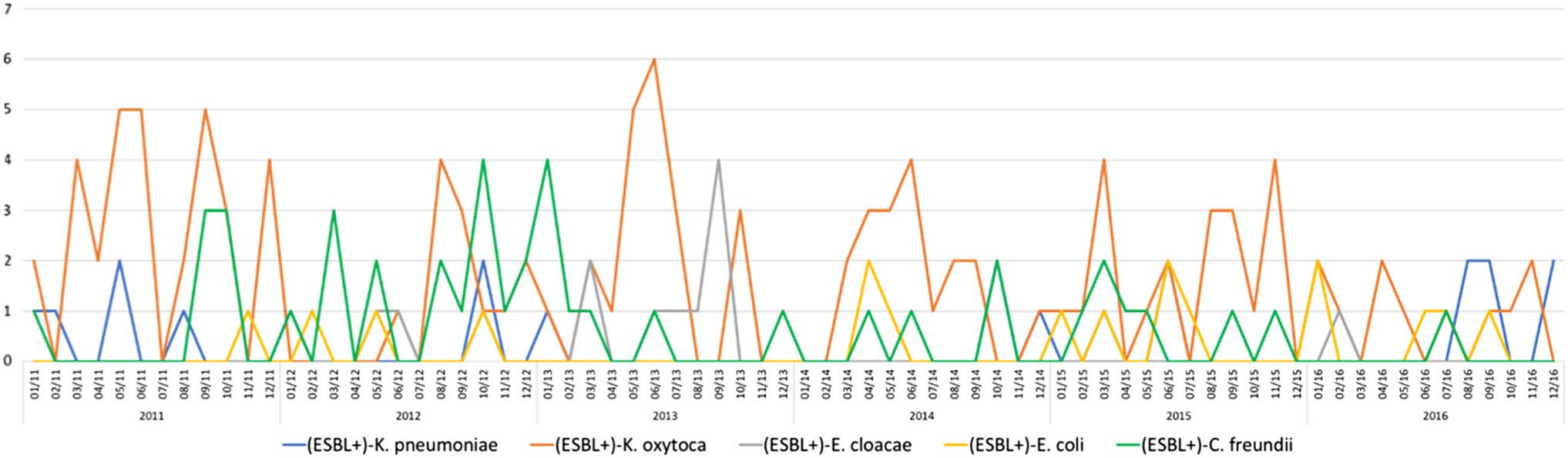
ESBL producing Enterobacterales in surveillance stool cultures of preterm infants ≤ 32 weeks of gestational age detected between 2011 and 2016

**Fig. 3:**
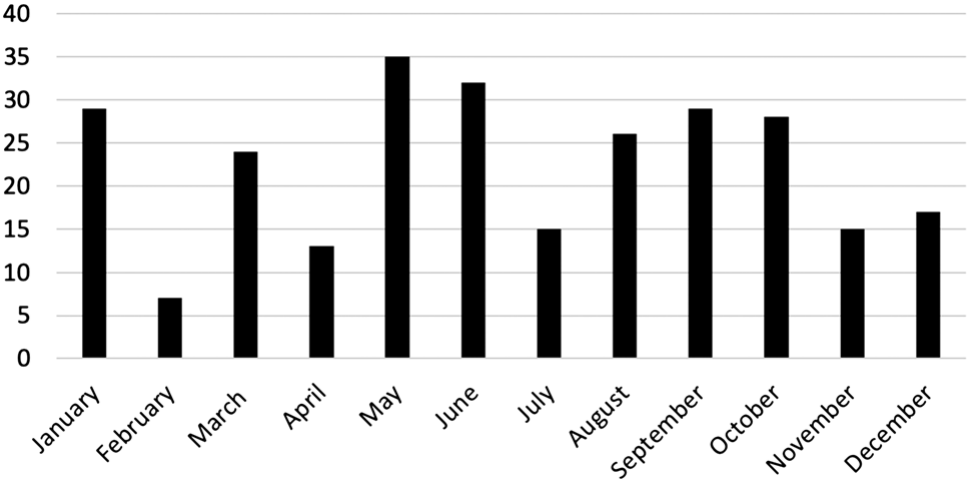
Monthly distribution of ESBL producing Enterobacterales from surveillance stool cultures of preterm infants ≤ 32 weeks of gestational age detected between 20,005 and 2016

We identified 217 inborn infants with a total of 270 ESBL-E and matched 217 controls between the years 2005 and 2016. Perinatal and neonatal data are shown in Table 1. The median time of detection of ESBL-E in stool surveillance cultures was 32 (range 4–233) days; and 110 (51%) infants were colonized at day 32 or before. Only nine infants (4.2%) were extremely late colonized at days 102, 103, 103, 108, 110, 119, 123, 124, 233, respectively). Forty-four ESBL-E positive cases (20.3%) were colonized with more than one species. In total there were ten different ESBL producing species, with ESBL-positve *K. oxytoca* being the most common one followed by *K. pneumoniae* and *Citrobacter freundii* (see Table 2). The majority of cases (203; 94%) were discharged being still colonized with ESBL-E. Changes over time are shown in Figs. 1 and 2. Between 2005 and 2009, the number of ESBL-E colonized infants ranged between 4.5 and 9.9% of all hospitalized preterm infants (≤ 32 weeks of gestational age) at the NICU and increased in the following years to rates between 23.6 and 32.0% (threefold increase). ESBL-producing *K. pneumoniae,* dominating in the early years of the study period, was replaced by ESBL-producing *K. oxytoca*. A seasonal distribution was not apparent (see Fig. 3). No bloodstream infection by ESBL-E was documented throughout the study period, also EOS and LOS did not change over time.

**Table 1:** *Perinatal and neonatal* data of 217 preterm infants ≤ 32 weeks of gestation (cases, faecal carriers of ESBL producing Enterobacterales) and 217 matched controls born between 2005 and 2016

	Cases	Controls	*p* value
Gestational age (weeks)	28 ± 2.7	28 ± 2.7	0.401
Birth weight	1202 ± 863	1155 ± 412	0.237
SGA	37 (17.1)	29 (13.4)	0.138
Multiple birth	85 (39.2)	47 (21.7)	< 0.001
Maternal age	30,4 ± 6,1	29.7 ± 6.0	0.127
Caesarean section	186 (85.7)	183 (84.3)	0.396
APGAR 1	6.5 ± 1.9	6.6 ± 2.0	0.222
APGAR 5	8.2 ± 1.2	8.2 ± 1.3	0.485
APGAR 10	8.8 ± 0.9	8.8 ± 1.0	0.276
UApH	7.29 ± 0.09	7.25 ± 0.52	0.149
Early onset sepsis	21 (9.7)	34 (16)	0.030
Late onset sepsis	35 (16)	31 (14)	0.290
Respiratory distress syndrome	194 (89)	201 (93)	0.121
Intraventricular haemorrhages	40 (18)	34 (16)	0.222
Periventricular echodensities (PVL I)	18 (8,3)	16 (7.4)	0.366
Cystic periventricular leukomalacia (PVL II-IV)	8 (3.7)	4 (1.8)	0.121
Bronchopulmonary dysplasia	21 (9.7)	19 (8.8)	0.370
Retinopathy of prematurity	32 (15)	34 (16)	0.395
Spontaneous intestinal perforation	4 (1.8)	8 (3.7)	0.123
Ileus	39 (18)	32 (15)	0.182
Death > 7 days	5 (2.3)	7 (3.2)	0.274

Data are given as *n* (%) or mean ± SD

*SGA* small for gestational age, *UApH* umbilical artery-pH

**Table 2:** ESBL producing Enterobacterales (*n* = 270) detected in 217 infants with single and multiple colonization between 2005 and 2016

ESBL producing Enterobacterales	Number	% *n* = 270	%* *n* = 217
*Klebsiella oxytoca*	125	46.3	57.3
*Klebsiella pneumoniae*	52	19.3	23.9
*Citrobacter freundii*	46	17.0	21.2
*Escherichia coli*	19	7.0	8.7
*Enterobacter cloacae*	17	6.3	7.8
*Citrobacter amalonaticus*	4	1.5	1.8
*Kluyvera species*	3	1.1	1.4
*Enterobacter aerogenes*	2	0.7	0.9
*Aeromonas species*	1	0.4	0.5
*Citrobacter farmeri*	1	0.4	0.5

Data are given as *n* (%)

*Percentage of 217 colonized infants

Ten cases were diagnosed as having any stage of NEC and two in the controls (*p* < 0.01). We identified seven infants with a definite diagnosis of NEC (stage ≥ IIa according to the modified Bell criteria) in the case group (see Table 3) compared to one infant in the controls (3.2 vs. 0.5%, *p* = 0.016, odds ratio-OR- 7.2, CI95% 1.2–42.3). NEC mortality rate (both cohorts) was 25% (2/8 NEC stage ≥ IIa cases). The association between ESBL-E and NEC was positive in four cases compared to one control (1.8 vs 0.5%, *p* = 0.089, OR 4.1, CI95% 0.45–36.6), findings not significant.

**Table 3:** Diagnosis of definite necrotizing enterocolitis in seven of 217 infants with the positivity of ESBL-E in stool surveillance cultures between 2005 and 2016

Case	wGA	BW	SGA	Apgar 1	5	10	UApH	ESBL-E	dNEC	dESBL	Death
m	26	1000	0	8	10	9	7.44	*K. pneumoniae*	11	15	No
m	26	898	0	8	8	9	7.33	*K. pneumoniae*	53	106	No
f	27	938	0	8	10	10	7.20	*K. pneumoniae*	14	14	No
m	28	1144	0	8	9	9	7.33	*K. oxytoca*	18	17	No
f	31	1256	0	4	7	9	–	*C. freundii*	64	31	Yes
f	31	1166	1	4	7	7	7.30	*C. freundii*	61	27	Yes
f	28	1130	0	5	8	9	7.31	*K. oxytoca, E. coli*	14	58	No
Mean	28	1076	–	6.4	8.4	8.9	7.32		34	38	*p* = 0.276

*wGA* weeks of gestational age, *BW* birth weight, *SGA* small for gestational age, *UApH* umbilical artery pH, *m* male, *f* female; *ESBL-E* ESBL positive Enterobacterales, *K* Klebsiella, *C* Citrobacter, *E* Escherich, *dNEC* day of life when definite diagnosis of NEC > IIa was done, *dESBL* day of life with first positive result of ESBL Enterobacterales in stool culture

## Discussion

Within this 12-year observation period epidemiological findings showed ESBL-producing Klebsiella spp. dominating all other ESBL producing Enterobacterales; and ESBL-producing *K. pneumoniae* being replaced by ESBL-producing *K. oxytoca*, with a dramatically threefold increase of the ESBL-E rate from maximum 9.9% (2005–2009) to 32% (2010–2015).

Infections caused by ESBL-E are of higher morbidity and mortality, so far also higher NEC-incidences caused by local outbreaks of invasive infections with ESBL producing pathogens have been reported in the literature [2, 3]. In an 8-year cohort of NICU patients, 339 of 1106 bacteremias were caused by gram-negative bacilli [19] The most frequent mechanism of resistance was ESBL production (67.1%), mainly by Klebsiella pneumoniae (59.6%). Among other previous antibiotic exposure to third-generation cephalosporin and carbapenem were identified as independent risk factors for multidrug-resistant gram-negative bacilli acquisition [19]. Thus our use of both antibiotics might be responsible for the marked increase of ESBL-E colonization at our NICU. Enterobacterales are among the early bacterial colonizers of the lower intestinal tract, and the relative abundance of some genera from this family such as *E. coli* and Klebsiella sp. are often increased in intestinal pathologies including inflammatory bowel disease and NEC [20]. The presence of a complex microbiota attenuates this response. A 16S rRNA gene-based analysis of fecal microbiota of neonates with and without NEC revealed a low diversity. The empiric and wide-spread use of antibiotics might play a crucial rule [21]. Persistently high levels of the facultative anaerobic Enterobacterales in fecal samples are considered to represent a delay in maturation of the gastrointestinal microbiota, along with a reduced level of anaerobes such as Bifidobacterium and Bacteroides. Normally, with time, the gastrointestinal microenvironment becomes depleted of oxygen, leading to a predominance of anaerobic organisms. A simulation of various colonization patterns to investigate the pathogenic potential of *K. pneumoniae* for the neonatal gut in a mouse model demonstrated that both, a nonexistent and a slowly acquired, but not a complex microbiota provided optimal conditions for this isolate to induce intestinal inflammation resembling NEC [21].

All together data let speculate about an association of ESBL-E positivity in stool surveillance cultures of preterm infants with a diagnosis of NEC. Including a twelve-year period of surveillance our matched case–control study did not show that there is evidence of an association between ESBL-E and definite NEC.

A very recent meta-analysis included eight studies that were available for quantitative synthesis including 106 NEC cases, 278 controls, and a total of 2944 microbiome (stool) samples [22]. The age of NEC onset was at 30.1 ± 2.4 weeks (mean ± SD) postconceptional age (*n* = 61). Microbial dysbiosis preceding NEC in preterm infants was characterized by increased relative abundances of Proteobacterales and decreased relative abundances of Firmicutes and Bacteroidetes [22]. Especially the use of antibiotics in newborns/preterm infants plays a crucial role in the pathogenesis of NEC and might be seen as a risk factor [23]

The total incidence of NEC in our study (both cohorts, 8/434) was 1.2%; and was at the lower limit compared to reports from the literature showing rates between 0 and 9.8% in studies without and rates between 0 and 16.7% with prophylactic use of probiotics [24]. Additionally, our NEC rate presented to be constantly low over the years when compared to the rate of 0.7% rate published 13 years ago [17].

More than a third of the cases were twins or triplets, a well-known finding regarding NEC [25]. So far no genetic predisposition for NEC is known, thus the role of multiple births as a risk factor for NEC still has to be elucidated. Bacterial translocation is supposed to play a role in the pathogenesis of NEC [26]. Interestingly, EOS was more common in the controls. Hence, EOS might not necessarily be an antecedent for NEC. NEC associated mortality rate was 25% corresponding with data reporting mortality rates of 20–30% in other studies [27, 28].

Especially maternal colonization with ESBL-E can lead to transmission of such pathogens to neonates, resulting in considerable morbidity in neonates [29]. Data from a Kenian hospital including 510 infants admitted without ESBL-E carriage demonstrated that more than half of the infants (238/55%) acquired carriage during their hospital stay [30]. The incidence of acquisition was 21.4% (CI95% 19.0–24.0%) per day. Thus, the rate of ESBL-E positively correlated with the number of known neonatal ESBL-E carriers and with the total number of neonates on the same ward. While ESBL-E were formerly restricted to hospitals, they have now spread to community settings [31, 32]. A Norwegian study reported that despite a low prevalence of ESBL-E carriage among pregnant women (2.9%), the maternal-neonatal transmission was 36%, thus the ESBL-E colonized mother was a risk factor for the newborn [34]. By means of the mother as the most important risk factor for colonization with ESBL-E among infants with low birth weight < 1500 g [35], authors postulated that it seems to be reasonable to screen mothers for ESBL-E [36].

Also a very recent systematic review and meta-analysis support these findings, with a pooled transmission rate of 19% (CI 3–35%) when mothers carry multiresistant Enterobacterales. Maternal screening for colonization with ESBL-E is not usual in Austria, so we do not have any data of that, but other studies from Austria demonstrate the problem of the rising percentage of ESBL-E in both, the hospital and the community [15, 32].

While strengths of the study include the carefully collected data using all available sources and the correct matching and the predefined criteria with regard to the association of ESBL-E and NEC, several limitations of the study have to be mentioned. First the retrospective design of the study is inferior to any kind of prospective study, and second, the low rate of NEC cases. Another limitation is the very theoretical background of our hypothesis based on microbiome changes by ESBL-E and the potential of Klebsiella spp. to induce inflammation and gut injury as tested in a mouse model that resembles NEC. Thus, larger studies with higher rates of NEC are needed to clarify a potential association with ESBL-E colonization of very preterm infants.

## Conclusions

We found no evidence that colonization with ESBL-E is a risk factor for the development of NEC. Rates of ESBL-E in stool surveillance samples at a NICU increased markedly during the study period.

## Data Availability

The datasets used and analyzed during the current study are available from the corresponding author on reasonable request.

## Abbreviations

EOS: Early-onset sepsis
ESBL-E: Extended-spectrum beta-lactamase producing Enterobacterales
LOS: Late-onset sepsis
NEC: Necrotizing enterocolitis
NICU: Neonatal Intensive Care Unit
OUT: Operational taxonomic units
Spp.: Species
